# Age‐associated changes in neuroimaging biomarkers in a non‐human primate model of cerebral amyloid angiopathy

**DOI:** 10.1002/alz70856_105980

**Published:** 2026-01-07

**Authors:** Suleiman Khan, Muhammad T Soliman, Sean Murray, Jakub Szabo, Michael Llanos, Hakyoung Kim, Ema Mir, Daniela Santoyo‐Alanis, Charles Kingsley, Jody Swain, Michele Marie Mulholland, William D Hopkins, Thomas Wisniewski, Jelle Veraart, Youssef Zaim‐Wadghiri, Henrieta Scholtzova

**Affiliations:** ^1^ NYU Grossman School of Medicine, New York, NY, USA; ^2^ Small Animal Imaging Facility, UT MD Anderson Cancer Center, Houston, TX, USA; ^3^ UT MD Anderson Cancer Center, Michale E. Keeling Center for Comparative Medicine and Research, Bastrop, TX, USA; ^4^ New York University Grossman School of Medicine, New York, NY, USA

## Abstract

**Background:**

Amyloid related imaging abnormalities (ARIA) have emerged as indicators of potentially serious side effects in therapeutics for Alzheimer's disease (AD). Squirrel monkeys (SQMs) are a unique non‐human primate (NHP) model for studying ARIA, due to their propensity of naturally developing age dependent cerebral amyloid angiopathy (CAA). Previously, we reported the sporadic occurrence of ARIA, manifesting as vasogenic edema (ARIA‐E) and cerebral microhemorrhage (ARIA‐H) in geriatric SQMs. Our initial studies demonstrated that quantitative *R*_2_* (1/*T_2_* *) mapping can serve as a noninvasive and effective imaging biomarker for monitoring gray and white matter (GM/WM) age‐related and region‐specific changes associated with SQM neuropathology. In the current project, we extended this work by echo‐averaging the multi‐gradient‐echo (MGE) dataset to generate 3D‐anatomical images used to assess in‐vivo brain volumetric changes across the SQM lifespan. Additionally, we examined a subset of SQMs using multi‐shell diffusion‐weighted‐imaging (DWI) to evaluate changes in microstructural tissue integrity.

**Method:**

SQMs underwent 2D‐*T*_2_‐w Turbo‐Spin‐Echo RARE/FLAIR MRI scans, alongside multi‐shell DWI. Diffusion metrics including mean diffusivity (MD), fractional anisotropy (FA), and mean kurtosis (MK) were calculated. Brain volumetrics were quantified as a function of age using the Jacobian of local deformation fields from in‐vivo MGE scans. Coronal brain sections were examined to characterize histopathological features of MRI abnormalities.

**Result:**

Multi‐shell DWI revealed abnormal propagation along the WM tract, which went undetected using conventional ARIA‐E criteria. The microstructural changes depicted on DWI were accompanied by alterations in MD, FA, and MK. Histologically, affected regions visible only in DWI showed differentiated pathology from conventional ARIA‐E regions, accompanied by dystrophic microglia, reactive astrocytosis, disruption of myelin integrity, and extensive fibrinogen extravasation. Comparing young to geriatric SQMs, deformation masks revealed age‐related volumetric decreases in PFC, putamen, nucleus caudate, and thalamus. When comparing middle‐aged to geriatric SQMs, negative volumetric correlations with age were observed in putamen and thalamus, WM regions, and to a lesser extent, PFC.

**Conclusion:**

This study advocates for the use of multiparametric MRI methodologies to achieve a more sensitive and specific interpretation of imaging abnormalities potentially linked to age‐associated cerebrovascular dysfunction. These findings highlight the SQM model as an ideal environment for studying neuroimaging biomarkers of AD and CAA.

